# Comment on: Gudu T, Stober C, Cope AP *et al*. Baricitinib set to join the Covid-19 therapeutic arsenal?

**DOI:** 10.1093/rheumatology/keab386

**Published:** 2021-04-25

**Authors:** Sujoy Khan

**Affiliations:** Immunology and Allergy, Queen’s Centre, Castle Hill Hospital, Cottingham, England

Dear Editor, The Editorial by Tania Gudu and colleagues [1] echoes the Adaptive COVID-19 Treatment Trial–2 (ACTT-2) study findings that showed JAK inhibitor baricitinib and remdisivir can provide a clinically meaningful benefit and safer management approach for treating severe COVID-19 [2]. A hypothetical but possibly real clinical scenario is patients with adult-onset Still’s disease (AoSD) presenting with a florid cytokine storm typical of autoimmune/inflammatory diseases [autoinflammatory–macrophage activation syndrome–hemophagocytic lymphohistiocytosis (AI-MAS-HLH)] but with CT imaging suggesting possible COVID-19 pathological lesions. Will treatment be according to protocols for AI-MAS-HLH or COVID-19–related-MAS-HLH? Patients with active AoSD will typically be on immunosuppressive drugs (such as MTX), and virally driven hyperinflammation can make clinical symptoms and inflammatory markers quite difficult to interpret, which may be further complicated by negative SARS-CoV-2 PCR on nasopharyngeal samples [3]. A triple combination therapy of baricitinib, remdisvir and dexamethasone has the potential to change the way we treat COVID-19-MAS-HLH [4].

Non-aerosol-generating procedures such as CT imaging (non-contrast CT chest followed by cardiac CT) has fundamentally changed the way we evaluate patients, but persistently negative nasopharyngeal PCR samples for COVID-19 may mean performing bronchoalveolar lavage for samples, which may prove quite challenging and which has its own limitations [5]. We would first need to take a systematic approach and use existing criteria to diagnose HLH, then reconsider our immunosuppressive strategies. Both conditions, suspected COVID-19-MAS-HLH and AI-MAS-HLH, would respond to immunosuppression, but the former will benefit from additional antiviral support, such as with remdisivir. Addressing the cytokine storm will determine the final outcome, and the triple combination of baricitinib, remdisivir and dexamethasone appears much safer than using transplant-related protocols routinely used for secondary HLH. Etoposide has serious side effects of lymphocytopaenia and derangement in coagulation parameters, which would complicate prognostic variables and patient recovery. Addition of remdisivir in such patients who are COVID-19 negative (but for whom imaging studies indicate COVID-19 is likely) will not necessarily add to the risk, while controlling viral replication if nasopharyngeal PCR samples were negative due to low viral loads or variable shedding. Successful clinical trials with the triple combination will also highlight the potential of added JAK inhibition, which is not in the current treatment pathways for HLH. 

*Funding*: No specific funding was received from any bodies in the public, commercial or not-for-profit sectors to carry out the work described in this article.

*Disclosure statement*: The author has declared no conflicts of interest.

## Data availability statement

Not applicable, as no new data generated for this article.

**Rheumatology key message**

- Baricitinib, remdisivir and dexamethasone is potential standard-of-care for treatment of COVID-19-suspected hemophagocytosis.
